# Recent Advances in the Plasma-Assisted Synthesis of Zinc Oxide Nanoparticles

**DOI:** 10.3390/nano11051191

**Published:** 2021-04-30

**Authors:** Gregor Primc, Katja Brenčič, Miran Mozetič, Marija Gorjanc

**Affiliations:** 1 Department of Surface Engineering, Jozef Stefan Institute, Jamova cesta 39, 1000 Ljubljana, Slovenia; gregor.primc@ijs.si (G.P.); miran.mozetic@guest.arnes.si (M.M.); 2 Department of Textiles, Graphic Arts and Design, Faculty of Natural Sciences and Engineering, University of Ljubljana, Aškerčeva 12, 1000 Ljubljana, Slovenia; katja.brencic@ntf.uni-lj.si

**Keywords:** plasma, synthesis, zinc oxide, nanoparticles

## Abstract

An overview of recent work on the low-temperature plasma-assisted synthesis of zinc oxide (ZnO) nanoparticles is presented and interpreted in terms of gas-phase and surface reactions with illustrated examples. The thermodynamical nonequilibrium conditions allow the formation of chemically reactive species with a potential energy of several eV, which readily interact with the Zn precursors and initiate reactions leading to the formation of nanoparticles or nanowires. The high-quality nanowires were synthesized from Zn powders only upon interaction with moderately ionized plasma in a narrow range of plasma parameters. This technique is promising for the synthesis of large quantities of nanowires with aspect ratios well above 10, but the exact range of parameters remains to be determined. Apart from the ex situ techniques, the ZnO nanoparticles can be synthesized by depositing a film of precursors (often Zn salts or Zn-containing organometallic compounds) and exposing them to oxygen plasma. This technique is useful for the synthesis of well-adherent ZnO nanoparticles on heat-sensitive objects but requires further scientific validation as it often leads to the formation of a semicontinuous ZnO film rather than nanoparticles. Both low-pressure and atmospheric plasmas are useful in converting the precursor film into ZnO nanoparticles despite completely different mechanisms.

## 1. Introduction

Metal oxide nanoparticles have attracted the attention of both the scientific community and users as their properties often differ significantly from those of bulk materials. Perhaps the most studied are titanium dioxide nanoparticles, followed by silica and zinc oxide nanoparticles (nano-ZnO) [1,2]. Nano-ZnO has multifunctional physiochemical properties, high photostability and a broad absorption band [3]. It is also considered to be nontoxic and it is stable at high temperatures [4]. Otherwise, ZnO has multifunctional properties such as photocatalytic self-cleaning and antimicrobial activity, ultraviolet (UV) protection, flame retardancy, thermal insulation, moisture management and electrical conductivity [5]. These properties enable ZnO to be used for development of superhydrophobic, antibacterial, antistatic, UV-protective and self-cleaning products, UV light sources, gas sensors, transparent electrodes in solar cells, spintronics devices and biosensors [5,6,7,8,9]. Numerous methods have been invented for the synthesis of ZnO nanoparticles, including wet-chemical methods, combustion of Zn-containing precursors, laser ablation, hydrothermal and electrochemical methods, and chemical vapour deposition [3,4,5,7,8,9,10,11,12,13,14,15,16,17,18]. Depending on the synthesis parameters, a variety of morphological shapes have been reported [13]. These include simple spherical particles, three-dimensional particles with more complex shapes, nanorods and nanowires. The latter can have aspect ratios greater than 10 or even greater than 100 [14,15,16,17,18,19,20,21,22,23]. The vast majority of ZnO nanoparticles have more spherical shapes since such a morphology represents the minimum surface energy and is the most thermodynamically favourable.

Plasma techniques for the synthesis of ZnO nanoparticles are becoming increasingly important as the thermodynamic nonequilibrium conditions allow the synthesis of nanoparticles with interesting morphological properties that cannot be achieved by other techniques. In addition, nonequilibrium conditions often allow chemical reactions to occur at a lower temperature than their thermal equilibrium counterparts, making them of interest for any application where the substrate cannot tolerate heating to high temperatures. An alternative to nonequilibrium plasma is the application of powerful discharges, which enable sustaining plasma at high temperatures. In the case of such hot plasmas, the chemical reactions leading to formation of nanoparticles are predominantly due to the high temperatures of both the gas and the solid particles. This paper reviews some recent work on the deposition of ZnO nanoparticles by plasma methods and explains the mechanisms, highlighting both the advantages and disadvantages of particular embodiments. Table 1 summarizes the plasma-assisted methods for synthesizing ZnO nanoparticles.

## 2. Plasma Methods for Synthesizing ZnO Nanoparticles

### 2.1. Low-Pressure Plasmas

**Figure 1:**
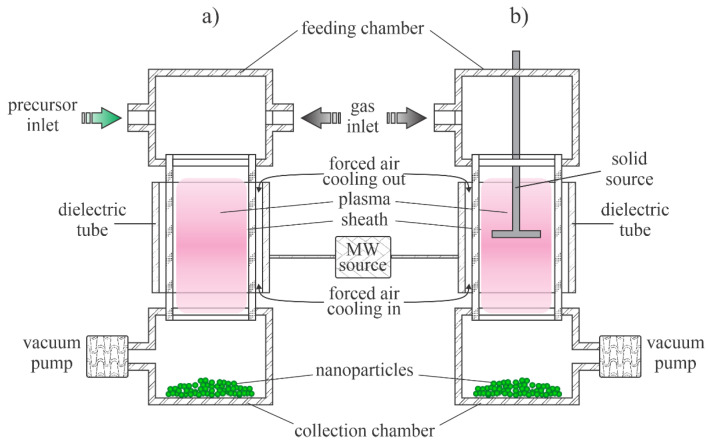
Schematic of the low-pressure microwave plasma system for nanoparticle synthesis: (**a**) with organometallic gaseous or powder precursor; (**b**) with solid precursor placed into the plasma reactor.

Low-pressure plasmas are popular for the treatment of solid materials due to the uniformity of plasma parameters over a rather large volume. The uniformity is due to the lack of the gas-phase collisions that lead to the loss of reactive species by neutralization of charged particles, the association of neutral radicals to stable molecules and relaxation of metastables in superelastic collisions [38]. Such plasmas can be sustained by various discharges, but the most popular are electrodeless discharges. The microwave discharges are particularly useful for the synthesis of zinc oxide nanoparticles. Plasma is sustained in a dielectric tube, which is mounted into a microwave cavity. A typical configuration is shown in Figure 1a. The dielectric tube is hermetical tightly mounted between the feeding and collecting chambers. The system is pumped with a suitable vacuum pump that enables evacuation as well as the removal of gaseous reaction products. The gas flow is in the direction from the feeding to the collection chamber. The dielectric tube is mounted into a metallic cavity which is powered with a microwave source. The standing waves in the cavity ignite the discharge in the dielectric tube. As soon as the plasma is ignited, the gas within the dielectric tube becomes conductive, so the skin effect prevents propagation of the electromagnetic field inside the plasma. The field is thus concentrated to the sheath between plasma and the dielectric tube, so it has a high amplitude, which is beneficial for the acceleration of electrons to a high kinetic energy. The fast electrons accelerated within the sheath collide with slow plasma electrons and transfer their energy in elastic collisions. The electrons inside the plasma thus assume a large temperature, often a few 10,000 K. Such electrons are capable of ionizing and dissociating collisions with the precursors. The precursors and reactive gases are fed continuously into the system. The nanoparticles are formed in the plasma volume and accumulate in the collection chamber. The pumping should be configured in such a way as to prevent significant removal of as-synthesized particles.

The configuration in Figure 1a is useful in cases of gaseous or powder precursors fed continuously into the plasma reactor. Other authors employed precursors placed directly in the plasma. Such a configuration is presented in Figure 1b. Any metallic object placed into the microwave field will be heated due to surface currents in electrically conductive materials stimulated by the oscillating electromagnetic field, so the precursor will melt and evaporate when using the configuration of Figure 1b.

Probably the most straightforward technique for synthesizing any metal oxide nanoparticle in the gas phase is to heat precursors in an oxidative atmosphere. The precursors (often Zn(CH_3_)_2_) are fed into a burner along with oxygen or water vapour. However, this technique may not lead to the best quality ZnO nanoparticles. To overcome the problem of optimal oxidation of Zn(CH_3_)_2_ precursors, Kleinwechter et al. [23] proposed using a microwave-driven plasma reactor, as shown schematically in Figure 1a. A typical discharge power was 60 W and a typical pressure was 30 mbar, and plasma was sustained in a mixture of 20 vol% O_2_ and 80 vol% Ar. The concentration of Zn(CH_3_)_2_ was varied between 700 and 1800 ppm. The nanoparticles were rather spherical with diameters of several nm and did not agglomerate. The particle diameter increased monotonically with the increasing concentration of the precursor. In the second experiment, the pressure was varied at a fixed precursor concentration, and the authors found an increasing particle diameter with increasing pressure. When the discharge power was varied, and other parameters were fixed, the nanoparticle dimension decreased with increasing discharge power. The authors found microwave plasma synthesis to be a suitable method to overcome the difficulties encountered in particle formation in a chemically heated rector—namely, instead of individual nanoparticles, a thin greyish film formed that strongly adhered to the substrate when the same gas mixtures were used to synthesize nanoparticles in a classical oven. According to the authors, the plasma system used by Kleinwechter et al. [23] enabled the choice between chemical and physical energy and a better understanding of particle formation processes. No details of the chemical reactions upon plasma conditions were given in [23], but there are numerous papers on microwave plasma behaviour in the range of pressures around 10 bar. A relatively complete insight into the gas-phase reactions in Ar-O_2_ plasmas sustained by microwave discharges was provided by Kutasi et al. [39]. The work presents scientifically sound simulations based on experimental observations. Indeed, current characterization techniques only allow the quantitative measurement of a few types of plasma species. Kutasi et al. [39] explained different gas-phase and surface reactions and concluded that the Ar-O_2_ mixtures in microwave plasma are always rich in O atoms. Dissociation of oxygen molecules occurs both at collisions of electrons from the high-energy tail of their distribution function as well as at collisions with Ar* metastables. The potential barrier for the oxidation of Zn(CH_3_)_2_ molecules with O atoms is much lower than for O_2_. The reaction efficiency depends on the concentration of two reactants. Kleinwechter et al. [23] reported the concentration of Zn(CH_3_)_2_ to be around 1000 ppm, which is much less than the concentration of O atoms, which is reported to be well over 10,000 ppm. Thus, the oxidation is efficient. This is because there are numerous channels for the dissociation of Zn(CH_3_)_2,_ and the electron energy required to subtract an H atom from the molecules is less than the dissociation energy of O_2_ molecules. The Zn(CH_3_)_2_ molecules form different radicals, and the oligomerization probably takes place at a pressure of several 10 mbar [3,4]. The clusters are heated under plasma conditions by heterogeneous surface reactions [40] so that their temperature remains high while they are in the plasma. This high temperature favours the complete oxidation of the clusters and thus the formation of highly crystalline ZnO. The clusters assume the floating potential the same as any other object immersed in a gaseous nonequilibrium plasma. A negative charge develops on the clusters while they are in the plasma, and the retarding electrostatic force suppresses agglomeration. As disclosed by Kleinwechter et al. [23], plasma synthesis ensures the high quality of ZnO nanoparticles and prevents their agglomeration.

**Figure 2:**
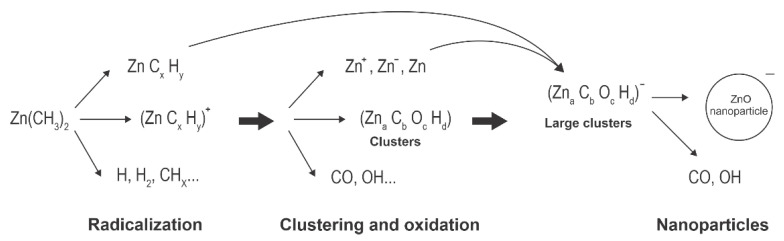
Schematic depiction of the complex reactions that can occur in gas mixtures of argon, oxygen and organometallic gasses under plasma conditions.

A schematic of plasma synthesis of ZnO nanoparticles using Zn-containing organic precursors is shown in Figure 2. The organometallic precursor forms radicals either by electron impact dissociation or quenching of Ar* metastables. The radicals are further split to form Zn atoms, which can also be excited or even ionized. Simultaneously, oxidation takes place, leading to the formation of molecules, such as OH, H_2_O, and CO, which are then associated with other atoms to form stable molecules (carbon dioxide and water). Radicals tend to associate with each other and form initial clusters. This effect was elaborated for other precursors (see the recent paper [41] and references therein). The initial small clusters adsorb radicals, and zinc atoms also condense on the surface, so the clusters grow. Simultaneously, they become negatively charged due to the attachment of slow plasma electrons. The negatively charged clusters attract positively charged species, causing further growth. Hydrogen and carbon, which are present in radicals and early stage clusters, readily interact with oxygen radicals (particularly O atoms that are in abundance in Ar-O_2_ plasma [39]) so almost pure ZnO is formed with a prolonged residence time. All surface reactions are exothermic. Hence, the cluster and nanoparticles’ temperature is well above the ambient temperature as long as they remain in plasma. 

Metallic vapour is also useful for synthesizing metal oxide nanoparticles. Rapid synthesis of ZnO nanoparticles using microwave plasma was reported by Subannajui [28]. Zinc swarf was placed in the centre of an alumina container and brought near to the microwave radiation source, as shown schematically in Figure 1b. The microwaves induced an internal potential that produced a very high field at the sharp peaks and edges of the Zn swarf. As a result, dense and hot plasma formed over the swarf, causing the material to melt and vaporize. The metal vapours condensed and oxidized in the region of the less intense plasma and the material was deposited on the surfaces facing the plasma in the form of ZnO nanoparticles. Even one second of such an aggressive treatment enabled deposition of some nanomaterials, but about 4 s was found to be the optimal treatment time for the growth of nanowires with a large aspect ratio. The early stage ZnO nanowires did not appear in the form of rods but as round and irregular long-ellipsoidal nanoparticles. Plasma treatment of a few seconds allowed the synthesis of nanowires with a rather uniform diameter of about 65 nm, but longer treatment times led to the deposition of films containing different shapes of ZnO. Subannajui [28] proposed a four-step mechanism. First, Zn atoms were sputtered or evaporated out of the swarf, and then they were oxidized, deposited and agglomerated on the substrate without catalysts. In the second step, a large number of ZnO nuclei were generated, and the Zn atoms were further oxidized and developed into larger ZnO nanoparticles. In the third step, the ZnO nanoparticles were stretched and became irregular long ellipsoidal particles. These long ellipsoidal particles tended to organize into the most stable state and become ZnO nanowires in the last step. A small peak corresponding to metallic Zn in the X-ray diffraction (XRD) spectrum indicated incomplete oxidation, but the oxide was found in the wurtzite structure of the ZnO nanowires. UV–Vis absorption spectroscopy showed good absorption in the UV region with a peak at about 365 nm.

A classical plasma-driven source of metallic atoms in the gas phase is a low-pressure discharge rich in energetic ions. The ions accelerate in the potential sheath next to the negatively biased electrode and cause sputtering. The removed metal atoms (including some ions) pass into the gas phase, where they can agglomerate if the pressure is high enough. This technique was elaborated by Kylian et al. [42]. This technique allows the synthesis of nanoparticles with selected diameters and compositions, depending on the discharge parameters, as recently shown by the same group [43]. The nanoparticles are always spherical when using this technique. The production of nanoparticles by this method is limited by the sputtering rate, so it does not allow mass synthesis. 

**Figure 3:**
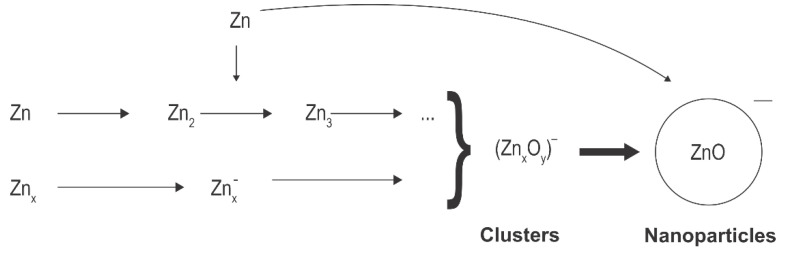
Schematic depiction of the reactions where zinc atoms or clusters are precursors.

Figure 3 shows schematically that the reactions in the case zinc atoms are precursors. As mentioned above, the source of atoms can be thermal evaporation [7] or sputtering [42]. Zinc atoms are partially excited and ionized, while any metallic clusters, likely formed by strong discharges [7], assume a negative surface charge due to the attachment of slow plasma electrons. Oxidation and agglomeration occur in a similar manner as when Zn(CH_3_)_2_ is used as a precursor. In all cases, the synthesized ZnO nanoparticles tend to have spherical shapes, and the diameter depends on the synthesis conditions, including residence time in plasma, pressure, the concentrations of the various gases, the discharge power, and specifics of the plasma reactor.

**Figure 4:**
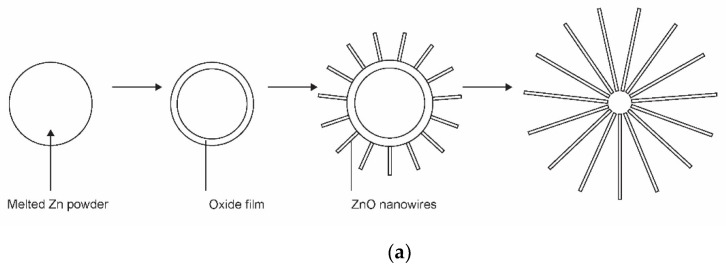

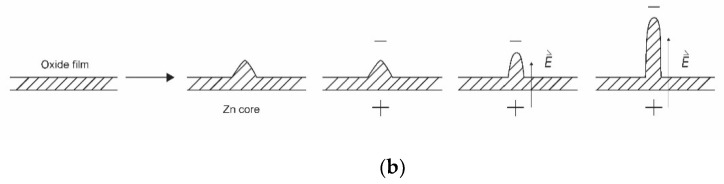
Schematic representation of the formation of nanoparticles with Zn powder as a precursor: (**a**) the formation of nanowires on the surface of metal powder; (**b**) the detail of the growth mechanism.

The application of gaseous precursors (organometallic compounds or Zn vapour) may not be economical or may not ensure the desired quantities of nanoparticles. The next option is to feed zinc powder into a plasma reactor. Hiragino et al. [26] synthesized nitrogen-doped ZnO nanoparticles using a medium-pressure gaseous plasma. The experimental system was almost identical to that presented in Figure 1a, except the microwave cavity was replaced with a coil connected to a radiofrequency (RF) generator. The discharge was concentrated to a rather small volume inside a coil which was coupled to an RF generator operating at a power of up to 30 kW and a frequency of 3.5 MHz. The gas pressure was about 200 mbar. The authors managed to keep the wall temperature of the reactor at 300 K by using water cooling instead of the forced air, as shown in Figure 1a. Zinc powder with a diameter of about 140 µm was used as the starting material. Based on the experimental details, the residence time of the powder in the hot plasma can be estimated to be about 0.1 s. Upon passing through the hot, but still nonequilibrium plasma, nanoparticles of different morphologies and a size of about 100 nm were synthesized. The aspect ratio of any nanoparticle was close to 1, and some agglomeration was observed, but the specific surface area of the synthesized ZnO materials was as large as about 20 m^2^/g. The materials were found to be useful for light-emitting diode (LED) applications. The schematic of the synthesis of zinc oxide nanoparticles from powder precursors is shown in Figure 4.

The kinetics of ZnO nanoparticle formation using Zn powder as a precursor is different from that shown in Figure 2 or Figure 3. The interaction of oxygen-containing plasma with metallic substrates was elaborated long ago, and one of the first reports on the synthesis of large aspect ratio nanowires was published as [44]. The metallic powder heats up to a high temperature when exposed to rather powerful plasma. Therefore, the powder quickly melts and sometimes even evaporates if kept in a powerful plasma for too long. The oxidation of the melted powder can lead to a variety of morphological shapes [45], ranging from nanowires with very high aspect ratios to cauliflower-like structures. The formation of nanowires on the surface of metal powder is illustrated in Figure 4a. The detail of the growth mechanism is shown in Figure 4b. The surface of a thin oxide film is never perfectly smooth but rich in humps. The plasma electrons accumulate at the top of the humps due to the Faraday effect. This creates a static electric field along the hump. The Zn^+^ ions enter the oxide layer and preferentially move in the direction of the electric field, i.e., along the hump, until they reach the tip of the hump, where they oxidize, thus lengthening the hump. A longer hump leads to further accumulation of the negatively charged electrons at the tip and thus even more extensive electro-diffusion of positive ions towards the tip. Finally, a nanowire with a large aspect ratio is formed. This mechanism, as shown in Figure 4b, works only under specific conditions, especially temperature. If the temperature is too low to enable diffusion of Zn^+^ ions through the oxide layer and on through the hump, little oxidation takes place. When the temperature is too high, thermal diffusion predominates, electro-migration is not as efficient, and structures with poor aspect ratios are formed. In one work, the range of useful parameters for the growth of silica nanoneedles was found only between 1800 and 1850 K [38].

Yet, another alternative for deposition of zinc oxide nanoparticles using a low-pressure gaseous plasma was reported by Yang et al. [25]. They used the configuration as shown in Figure 1b with some modifications to synthesize ZnO nanoparticles from ZnCl_2_ powder under low-pressure conditions. Instead of using microwave heating, a furnace was placed on a glass discharge tube to melt and heat the ZnCl_2_ powder to a temperature of about 350 °C, which allowed evaporation under controlled conditions. The vapour was directed toward a substrate by drifting a mixture of argon and oxygen in the direction from the precursor to the substrate. Weakly ionized gaseous plasma was generated in the discharge tube by an inductively coupled RF generator operating at a frequency of 13.56 MHz and a power of 100 W. The coil had the same function as the microwave cavity in Figure 1b. At plasma conditions, the vapour was partially atomized and the Zn atoms condensed on the substrate surface. The high concentration of oxygen atoms in the discharge tube allowed rapid oxidation of the deposited zinc. The treatment time was 15 min. The ZnO nanoparticles formed dense pillars with a typical diameter of about 0.4 µm and a length of several µm. XRD characterization confirmed the hexagonal ZnO structure with the preferential growth of ZnO was along the c-axis, which was confirmed by the transmission electron microscope (TEM) observations. Neither a cubic ZnO phase nor ZnCl_2_ were found in the prepared product. Without plasma, only ZnCl_2_ was found on the substrate. This work clearly demonstrates the advantages of plasma conditions. The authors suggested several mechanisms occurring in the gas phase and at the surfaces and emphasized the importance of metastable Ar in ZnCl_2_ dissociation. The authors also reported that the addition of O_2_ to the gaseous plasma caused an enhancement of ZnCl_2_ dissociation. Optical emission spectroscopy was used to characterize the plasma; the radiative transitions of Zn atoms were significantly suppressed even with a small addition of oxygen to argon. The intensity of the Zn atomic lines decreased with increasing concentration of oxygen in the Ar-O_2_ mixtures. The authors explained this by extensive oxidation of Zn in the gas phase. Therefore, the reaction mechanisms in the gas phase follow the initial case shown in Figure 3.

Sputtering or thermal evaporation using nonequilibrium low-pressure gaseous plasma may not be the most efficient atom sources due to limited power density and thus evaporation rate. Low-impedance discharges perform better as long as the evaporation intensity is the merit. Shanenkova et al. [7] reported the plasma synthesis of zinc oxide in an extremely short time process (duration less than 1 ms) using an electric discharge zinc-containing plasma jet flowing into an oxygen atmosphere. They used a home-built, high-power, arc-like plasma device operating in pulsed mode with the maximum discharge current of nearly 10^5^ A. The total energy of each pulse was nearly 30 kJ. The arc chamber was evacuated before igniting the discharge. The powered electrode was made of zinc, which vaporized upon the discharge conditions. Plasma was therefore sustained in metallic vapour. A shock wave formed during each discharge, and the plasma, rich in ionized metal vapour as well as very small Zn droplets, moved away from the main electrode at supersonic speed. The jet gradually cooled, allowing the clustering of Zn atoms in the gas phase, as shown schematically in Figure 4. The clusters oxidized in the oxygen-containing postdischarge chamber and grew by adsorption of Zn atoms. Complete oxidation of nanoparticles was observed. Various morphological shapes other than nanowires were found by TEM. Details about this unique device used for the synthesis of nanoparticles with typical dimensions of around 100 nm were reported in another work of the same group [29].

### 2.2. Hot Atmospheric-Pressure Plasmas

**Figure 5:**
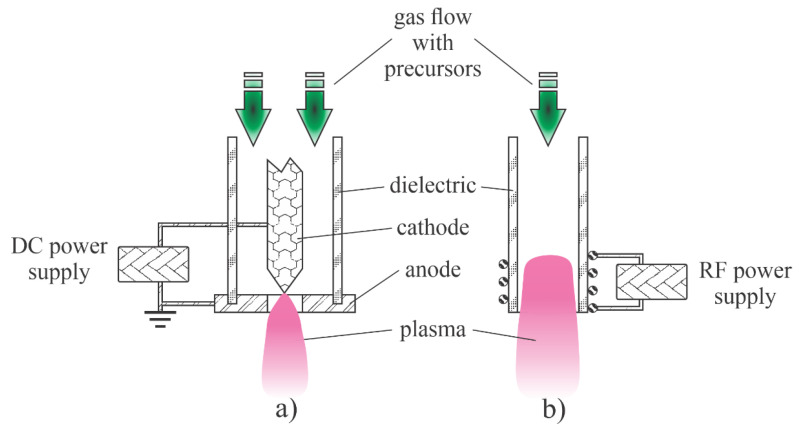
Direct current (DC) plasma arc (**a**) and radio frequency (RF) plasma torch (**b**).

The thermalization of plasma radicals and charged particles in the gas phase is a quick process at atmospheric pressure. Charged particles are created in the volume of a high electric field where the electrons gain enough energy for multiplication at ionization collisions with gaseous molecules or atoms. Outside this volume, the neutralization of charged particles, the association of radicals with stable molecules and relaxation of excited particles is so efficient that the density of reactive species is marginal. A couple of distinguished discharges are used for sustaining plasma at atmospheric pressure: the low-impedance electric arcs (often called plasma torches) and high-impedance discharges such as corona and dielectric-barrier discharges. A schematic of two devices useful for synthesizing ZnO nanoparticles using a low-impedance discharge is shown in Figure 5. Figure 5a shows a typical arc plasma powered by a low-impedance direct current (DC) discharge. The precursor, either organometallic or metal powder, is fed in the gas before the discharge zone or into the plasma plume. In the case of Figure 5b, the plasma plume is sustained with an electrodeless discharge, typically powered with an RF generator of frequency between 10 and 1000 kHz. In both cases, the gas temperature inside the plasma plume is around 10,000 K.

Lee et al. [37] developed a technique to synthesize high-quality ZnO nanoparticles by passing a relatively spherical zinc powder through an atmospheric pressure plasma. The plasma was sustained in air or a mixture of nitrogen and oxygen by a microwave discharge at a frequency of 2.45 GHz in a reaction chamber similar to the configuration of Figure 5b except that the microwave (MW) cavity was too small (and the electromagnetic field so dense) to enable sustaining plasma even at atmospheric pressure. The density of charged particles in the core plasma was about 10^19^ m^−3^. The kinetic temperature of the neutral gas reached 6500 K, similar to the arc discharges of Figure 5. The zinc powder melted under these harsh conditions and solidified after leaving the volume of the intense plasma. The powder was also oxidized during the plasma treatment, and the resulting ZnO particles took on interesting morphologies that depended on the details of the processing parameters. When compressed air was used, ZnO nanowires and tetrapods were synthesized. The concentration of nanowires was much larger than other morphological forms. Dense nanowires with diameters of about 100 nm and lengths of a few µm grew from particles with smaller aspect ratios under plasma conditions. The synthesis mechanism was similar to that illustrated in Figure 3. Both nanowires and tetrapods were single-crystalline. When the compressed air was replaced by high purity synthetic air or pure oxygen, a large number of nanoparticles grew from zinc powder, but they had more complex crystalline structures. The differences were explained by the presence of water vapour and some gaseous impurities in the compressed air, which were not present in pure gases. Optical absorption in the UV range (probed between 200 and 400 nm) was large for all morphological forms, but absorption in the visible range was found to be much larger for nanowires than for tetrapods. The photoluminescence of all ZnO materials peaked in the range between 350 and 360 nm.

A standard configuration of the atmospheric-pressure electric arc is shown in Figure 5a. Such a configuration of the discharge power 70 kW was used by Ko et al. [35] to synthesize ultrafine ZnO nanopowders. They used either pure nitrogen or a mixture of nitrogen and argon as carrier gases and commercial zinc powders as precursors. The powder melted and almost fully evaporated in the powerful discharge and the metallic vapour condensed when leaving the hot plasma. Oxidizing impurity gases assured for simultaneous oxidation of the nanoparticles upon solidification. Most synthesized nanoparticles were spherical of a typical diameter of several 10 nm, but more complex geometries were also found. The aspect ratio of such morphological features was not large, though. The production rate was over 1 kg/h. The configuration in Figure 5a therefore enables the rapid synthesis of rather large quantities of almost spherical ZnO nanoparticles.

A similar device was also used by Murali and Sohn [36] except that they used zinc nitrate powder of typical dimension 50 µm as the precursor. The discharge power was 15 kW and the Ar the carrier gas. As in the case of Ko et al. [35], the rather large powder melted and evaporated while passing the discharge zone, and the vapours condensed in the postglow region to form almost perfectly spherical nanoparticles on various diameters between about 10 and 50 nm. The formation of the nanoparticles followed the scheme presented in Figure 3. A more thorough explanation of the method is presented in the recent paper [46] of the same authors. An earlier review on the plasma arc synthesis of various nanoparticles using the configuration in Figure 5a was published by Seo and Hong [47].

A DC plasma source as shown in Figure 5a was also applied by Yu et al. [34]. Unlike the previously cited authors, Yu prepared an aqueous solution of zinc acetate and nitrate and sprayed the liquid into the plasma plume. The discharge was sustained in a mixture of argon and hydrogen. As a result of the spraying, the water quickly evaporated and dissociated upon plasma conditions, so the plasma was also rich in OH radicals as well as O atoms and ions. A substrate was placed in the agglomeration zone (a few cm from the plasma plume), so the nanoparticles were still hot upon impinging the substrate. Such a configuration was found useful for the synthesis of ZnO thin films with dense nanoparticles in different morphological shapes. The authors provided a schematic of the solution precursor plasma spray technology and the potential clusters from the different zinc solution precursors. Using pure Zn-acetate, the nanostructured morphologies were clearly observed. The films exhibited hierarchical microstructures composed of sea-urchin- and aloe-vera-like structures. The ZnO samples prepared from pure Zn-nitrate solutions exhibited classic porous microstructures composed of agglomerates of irregular particles or flatten splats. The clustering at the edge of the plasma plume where the temperature was reasonably low followed the reaction presented schematically in Figure 2, but the rich morphology observed in some samples indicates that the growth of the oxide nanostructures only occurred at a cooler zone of the discharge or even on the surface of the sample—namely, the morphology also exhibited nanospikes of very large aspect ratios, which is typical for the growth of the oxide film at conditions where the electro-migration prevails (Figure 3). In any case [34], clearly demonstrates that the morphology of ZnO surface films depends largely on small details in the composition of the sprayed precursors, which in turn influence the behaviour of plasma—in particular, the concentration of reactive plasma species in the early afterglow zone of the arc discharge.

The proximity of substrate to the edge of the hot plasma plume in the configuration shown in Figure 5b was also demonstrated by Wallenhorst et al. [48]. In fact, they developed a technique for deposition of nanostructured films on different substrates in the continuous mode. The substrate passed through the plasma jet at a speed of 4–5 cm/s. The working distance between the edge of the plasma plume and the substrate was about 2 cm. The discharge was ignited at the DC voltage of 15 kV and sustained at the effective voltage amounts of 2–3 kV. The input power reached a maximum of 2 kW, where high voltage pulses with a pulse duration of 5–10 µs and a pulse repetition rate of 50 kHz are applied. Zn powder of particle size about 13 µm was injected continuously into the plasma plume. A semicontinuous film rich in dense crystallites of ZnO was observed on the sample surfaces. Despite the rather large size of precursor particles, an almost closed basic layer of very small, nanometre-sized particles in between larger particles and/or agglomerates was detected. The existence of particles at the nanoscale was explained by the melting and vaporization of initial particles within the hot plasma zone and selective deposition of an almost closed layer of small particles and some agglomerated big clusters. Due to the relatively low power, the pulsed character of the discharge and the short residence time in the plasma plume caused the rich morphology, as observed by Yu et al. [34].

The configuration illustrated in Figure 5b was used by Yoo et al. [24]. They used argon as the carrier gas and zinc oxide powder with an average diameter of 1 µm. Despite the high discharge power up to 60 kW and the small size of the precursor powder, some unmelted powders were found in the collection chamber. Most powders, however, melted and evaporated in the hot plasma. The vapours and perhaps droplets of melted ZnO condensed in the postglow and formed spherical nanoparticles of diameters of several 10 nm. Some experiments were also performed at a somehow lower pressure (about 0.5 bar) and other morphological shapes were observed. No features of a high aspect ratio were detected by dropping the precursors through the discharge of Figure 5b though. The results reported by Yoo et al. [24], therefore, confirm the hypothesis that the nonequilibrium conditions are needed for plasma synthesis of metal oxide nanoparticles of high aspect ratios. As mentioned above, the mechanism illustrated in Figure 3 performs well only when a significant deviation from thermal equilibrium occurs. Otherwise, the spherical shape predominates since the spheres are the most thermodynamically stable forms of small particles. In another set of experiments, the authors managed to prolong the residence time of precursors in the early postdischarge region [24]. When using the extreme conditions (residence time of several seconds), much richer morphological shapes were observed. In fact, nanoflowers consisting of many hexagonal nanorods having lengths of a few μm and diameters of several 100 nm. Since the initial nanodroplets passed the extended growth region with enough residence time and abundant resources, the foundational nucleation seeds were formed and combined with floating ZnO molecules to assemble nanocrystal systems following the scheme presented in Figure 3.

### 2.3. Cold Atmospheric-Pressure Plasmas

**Figure 6:**
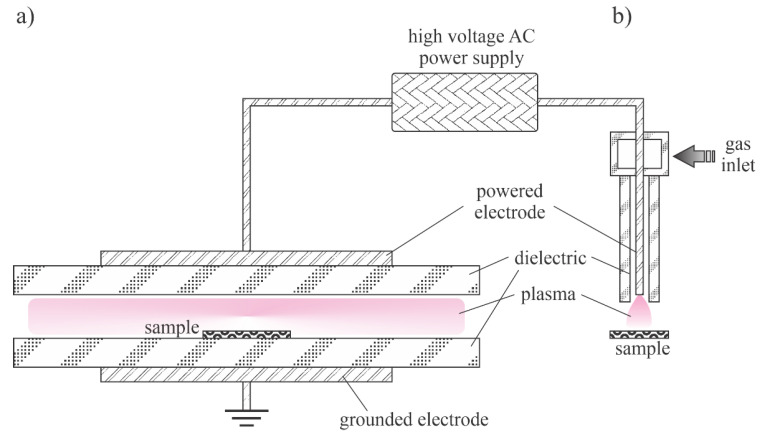
Schematic of a device useful for sustaining nonequilibrium plasma at atmospheric pressure. (**a**)—Plane parallel and (**b**)—jet configurations.

ZnO nanoparticles are often required as desired surface finish of other materials. In such cases, the adhesion of the nanoparticles synthesized by any technique described above may be insufficient. If adhesion is a problem, one solution may be to deposit the precursor on the substrate, followed by plasma treatment. The gaseous plasma should be cold enough to prevent melting or other thermal damage of the substrate. Even a brief exposure of a zinc plate to nonequilibrium plasma can cause a significant modification of the surface morphology. Zhang et al. [30] reported a simple method to fabricate ZnO nanosheet-assembly film by cold plasma sustained in ambient air. They used a double dielectric barrier discharge (DBD) to sustain the gaseous plasma. The device is illustrated in Figure 6a. An alumina ceramic plate backed by an electrode served as a holder for a zinc plate. Another plate was made of glass and provided with a counter-electrode. The electrodes were connected to a 5 Hz sine-wave power generator operating at a voltage of 12 kV. A filamentary discharge occurred in the volume between the electrodes, and gaseous plasma interacted with the zinc plate. A variety of zinc nitrates were found on the surface of the zinc plate by XRD analysis. Some samples were then heated to about 250 °C to allow the formation of ZnO in the form of nanowalls with a thickness of a few 10 nm and a distance between adjacent nanowalls of a few 100 nm. The unique morphological features were explained by localized heating of the zinc plate. The very low-frequency discharge caused streamers of fast electrons to hit the zinc surface. The electron streamers caused localized ionization and dissociation of gaseous molecules. The energy dissipated locally on the zinc plate surface was high enough to cause both oxidation and heating, which led to a locally high temperature and thus high mobility of atoms at the surface and below the surface. The presence of water vapour and reactive nitrogen species caused the formation of complex compounds.

A thin film of zinc-containing material can also be converted to ZnO nanoparticles upon treatment with filamentary DBD in the configuration illustrated in Figure 6b. A nitrogen DC-pulsed atmospheric pressure plasma jet (APPJ) was used by Lee at al. [33] to synthesize platinum-decorated ZnO nanoparticles. Chloroplatinic acid dissolved in isopropanol and zinc acetate in deionized water were mixed, stirred well and spin-coated onto fluorine doped tin oxide (FTO) substrates. The thin film was then treated with APPJ. The plasma treatment allowed the conversion of the coated film into high-quality ZnO nanoparticles decorated with metallic platinum. The effects were explained by rapid chemical reactions induced by the plasma treatment, as well as by thermal effects. The same coatings were also cured in an oven, and the plasma-treated materials exhibited higher quality, which was explained by the interaction of plasma radicals of high excitation energy with the deposited film. The materials were found to be useful as counter electrodes of dye-sensitized solar cells.

**Figure 7:**
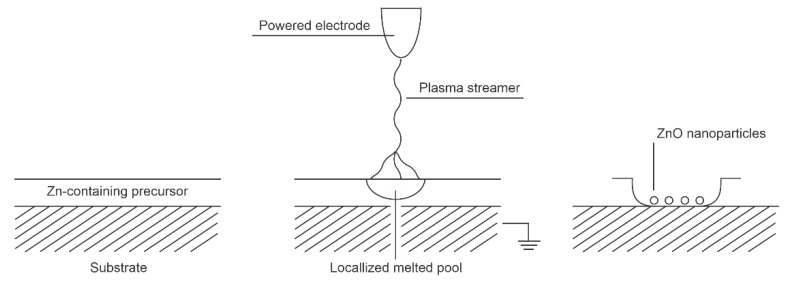
Interaction between low-frequency atmospheric pressure discharges and Zn-containing surface film.

The low-frequency alternative current (AC) or pulsed DC discharges at atmospheric pressure are not able to sustain a homogeneous plasma as the lifetime of the reactive species at 1 bar is nearly 1 µs—much shorter than the duration of a low-frequency AC discharge, let alone a DC-pulsed discharge. Such discharges result in the formation of one or a few current pulses due to the formation of electron streamers. A bunch of such streamers is often called a filament. The density of charged particles in a streamer is usually greater than in a continuous low-pressure plasma (Figure 1). Therefore, for a short time, extensive surface chemistry takes place at a small spot on the material when it interacts with the streamer. These spots are locally heated to a high temperature, which is often sufficient to convert a continuous film of the deposited precursor to ZnO nanoparticles, despite the rather low average temperature of the substrate. Figure 7 illustrates the effect of a streamer on the substrate surface. Numerous streamers affect larger surface areas, but the nanoparticles may vary in morphology or even composition from spot to spot, depending on the intensity of each streamer. The nanoparticles can adhere well to the substrate as not all of the precursor needs to be oxidized.

**Figure 8:**
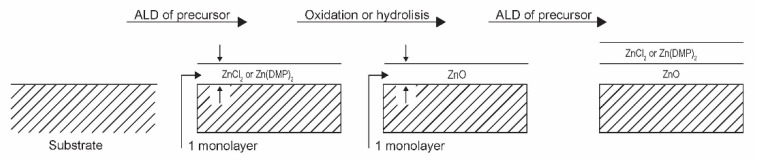
Oxidation of extremely thin layers of Zn-containing precursors.

An advanced method for low-temperature oxidation of Zn-containing organic precursors is based on atomic layer deposition (ALD). This method is promising in microelectronics as only the surface atoms should chemically interact with the gas environment, so, at least theoretically, the oxidation could be carried out at room temperature. Mai et al. [27] reported the deposition of uniform, smooth, stoichiometric, and highly transparent ZnO films from organometallic precursors. They focused on bis-3-(N,N-dimethylamino)propyl zinc (II) ([Zn(DMP)_2_]). The authors used the plasma-assisted atomic layer deposition method under low-pressure conditions. The organometallic precursors were heated to an elevated temperature and condensed on silicon wafers in the form of a very thin film (about one monolayer). The deposition time was about 0.1 s. Oxygen was then injected into the experimental system, and the plasma was ignited, allowing reactive oxygen species to completely calcinate the extremely thin organometallic film. The plasma was powered with a capacitively coupled RF discharge at 13.56 MHz and nominal discharge power of 200 W. The oxygen was introduced into the chamber by pulses of 500 ms, in which the plasma was ignited for 150 ms. Such a treatment time was long enough for the calcination of the monolayer of the organometallic precursor. The process was repeated several times until a ZnO layer of thickness several 10 nm was formed on the substrate. Incomplete calcination was observed at shorter plasma treatment times, indicating the importance of plasma species on the calcination process. Films of good quality were observed even at substrate temperature as low as 60 °C. Therefore, this technique is suitable for the deposition of ZnO films on heat-sensitive objects due to the favourable calcination temperature. The chemical reactions between reactive oxygen species and the deposited thin layer of organometallics are apparently extensive enough to allow complete oxidation over a time scale of well below 1 s. However, the need for numerous repetitions of deposition and calcination phases may be a drawback. The procedure is shown schematically in Figure 8.

Other precursors have been used in the atomic layer deposition of ZnO films. For example, Kopalko et al. [6] used zinc acetate (Zn(CH_3_COO)_2_) as a precursor but reported several obstacles that prevented the deposition of a uniform film in a reasonable time. The authors avoided oxidation of the precursor film by oxygen plasma and preferred hydrolysis. As a result, the required temperature to perform calcination was found to be around 300 °C. They also investigated zinc chloride as a precursor film, but the desired temperature to achieve the hydrolysis in a reasonable time was 500 °C. In any case, the resulting morphology of ZnO materials obtained by oxidation or hydrolysis of very thin films of Zn-containing precursors leads to the formation of at least semicontinuous films instead of high-quality ZnO nanoparticles.

### 2.4. Plasma Treatment of Liquid Precursor

**Figure 9:**
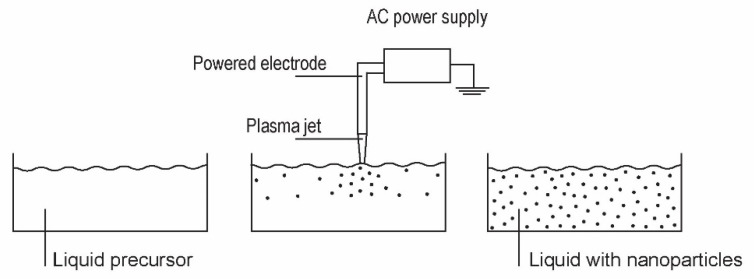
A typical setup for the synthesis of ZnO nanoparticles in liquids.

Zinc oxide nanoparticles can also be synthesized by plasma treatment of liquid precursors. Shutov et al. [3] developed a technique for the synthesis of powdered zinc oxide using plasma sustained over an aqueous zinc nitrate solution prepared by dissolving analytical grade Zn(NO_3_)_2_·x·6H_2_O in distilled water. The distance between the electrodes and the liquid solution was several cm, and the treatment time was 10 min. As a result of such indirect plasma treatment, a colloidal solution was formed in the anode compartment of the electrolytic cell. A precipitate was formed at the bottom of the cell. XRD characterization of the synthesized particles revealed a mixture of hydroxide nitrate hydrate, zinc hydroxide nitrate, zinc hydroxide and zinc oxide. The concentration of ZnO was a few percent, and the particles of a typical size of a few µm had a low aspect ratio. Plasma-assisted synthesis of zinc oxide nanomaterials was also reported by Ananth et al. [31]. A plasma jet was generated with a DBD discharge operated in the air at ambient pressure. The discharge was powered with a pulsed AC generator at a voltage of about 500 V and a current of 100 mA. An aqueous solution of zinc nitrate hexahydrate was used as a precursor. The plasma plume was about 1 cm away from the liquid surface so that the solution was treated by radicals in the flowing afterglow and any radiation arising from the glowing plasma that was not absorbed in the air. The plasma radicals were allowed to interact with the liquid for one hour. The temperature of the liquid remained below 30 °C throughout the treatment. A significant portion of the water had evaporated before the treatment was complete, revealing interesting interaction mechanisms. The plasma radicals allowed the formation of nanoparticles with various morphologies, including nonuniform ZnO cubes with a typical size of about 300 nm, nanorods with a length of up to about 1 µm and an aspect ratio of about 10, nanopillars with a length of just over 1 µm and an aspect ratio of about 3, flower-like structures with a typical size over 1 µm, and various agglomerates. Other morphological forms were observed when the precursor was replaced by sulphate heptahydrate or zinc chloride. In the case of sulphate, layered structures were observed. The authors suggested a number of processes that could be involved in the synthesis of the nanoparticles by precipitation during the plasma treatment, such as the dissolution of the plasma ions in the solvent and the recombination leading to the release of corresponding gases, which requires sufficient activation energy, or a sufficiently strong electric field. The XRD analyses revealed predominantly hexagonal wurtzite ZnO with some defects. The antibacterial activity of the ZnO nanomaterials showed strong shape-dependent properties and exhibited a high inhibition effect against several types of bacteria. The synthesis of ZnO (and other) nanoparticles from liquids is shown in Figure 9.

Plasma-induced synthesis of ZnO nanoparticles from liquid precursors was also reported by Tsumaki et al. [32]. They used atmospheric pressure plasma powered by a DBD discharge, similar to that shown in Figure 6b. The AC voltage source operated at a voltage of about 15 kV and a frequency of 29 kHz. Helium served as the process gas, and small droplets of zinc-containing precursors were fed into the discharge zone. The droplets contained an aqueous solution of zinc acetate dehydrates. The droplets dimensions depended on the atomizer setting, but a typical diameter was about 1 µm. As a result of the plasma treatment, a large number of ZnO nanoparticles were formed as they passed through the plasma zone. Both spherical particles and flakes formed upon plasma treatment, and a typical dimension was in the submicrometre range. The XRD showed a wurtzite structure. The diameter of spherical particles slightly depended on the concentration of precursor in the aqueous solution. In general, the particle diameter increased with increasing concentration, but the effect was not dramatic. The plasma was characterized by optical spectroscopy. The high-resolution spectrum of OH emissions with a band head at about 309 nm was recorded to estimate the rotational temperature of OH radicals, which was expected to be nearly equal to the gas kinetic temperature. The scientifically pristine theoretical spectrum was simulated and compared to determine the gas temperature in the glowing plasma, which was found close to 1000 K. A significant amount of water therefore evaporated, so the processes involved upon passing the droplets through plasma may be a combination of reactions, as illustrated in Figure 2 and Figure 9.

## 3. Conclusions

The literature review shows various methods for the synthesis of ZnO nanoparticles using nonequilibrium gaseous plasma. The precursors can be added directly into the gaseous plasma. In such cases, the plasma reactors operate at low-pressure conditions to avoid agglomeration of the synthetized ZnO nanoparticles. An interesting alternative is to apply an atmospheric-pressure plasma jet and feed precursors dissolved in small water droplets. In such cases, the water evaporates at least partially under the plasma conditions and the precursor transforms into ZnO nanoparticles. The water solutions in an appropriate container can be exposed to the atmospheric-pressure gaseous plasma, and the nanoparticles form without the need for water evaporation. However, controlling the morphological properties of ZnO nanoparticles synthesized in liquids remains a scientific challenge. Ex situ synthesized nanoparticles may not adhere well to a substrate. To overcome the problem of insufficient adhesion of ZnO nanoparticles to substrates, a precursor was deposited on a substrate and then treated with gaseous plasma. This technique often enables good adhesion of the ZnO film formed from the precursor, but the zinc oxide may be in the form of a semicontinuous film rather than adequately distributed nanoparticles. An alternative is the application of hot plasmas sustained by low-impedance discharges at atmospheric pressure. Precursors melt in the plasma plume. If the substrate is placed a few cm from the region of hot plasma in the direction of the gas jet, the nanoparticles remain hot enough to stick well on the substrate and form a film that may have a rich morphology. Decoration of heat-sensitive substrates with well-adhered uniformly distributed ZnO nanoparticles of suitable size and morphology, however, remains a scientific challenge.

## Figures and Tables

**Table 1:** Summary of plasma-assisted methods for synthesizing ZnO nanoparticles.

Plasma System/Discharge Type		Discharge Parameters	Gas	Zn-Precursor	Substrate for Deposition	ZnO Morphology	Size	Other ZnO Properties	Ref. No.
Low-pressure	Radiofrequency (RF) inductively coupled thermal plasma	p = 350, 650 and 700 Torr P = 28–31 kW f = 1–3 MHz	Ar as carrier gas	ZnO powder	N/A	polygonal at 350 Torr, round at 650 Torr, flower-like with hexagonal nanorods at 700 Torr	60–70 nm at 350 Torr, 30–50 nm at 650 Torr, 200–500 nm in diameter with 3–6 nm rods at 700 Torr	good crystal quality and optical property	[24]
	RF inductively coupled plasma	p = 1 Pa P = 100 W f = 13.56 MHz t = 15 min	Ar/O_2_	ZnCl_2_ powder	Si wafers	cone-sharp vertically grown	N/A	hexagonal ZnO structure	[25]
	RF plasma torch	p = 150 Torr P = 30 kW f = 3.5 MHz	Ar/O_2_ for ZnO Ar/air for N-doped ZnO	Zn powder 100–180 µm	glass	spherical	Zn powder feed rates enlarge particle size	hexagonal ZnO wurtzite structure	[26]
	RF plasma enhanced atomic layer deposition	p = 5 × 10^−4^ mbar P = 200 W f = 13.56 MHz T_deposition_ = 60–220 °C t = 50–150 ms	O_2_	bis-3-(N,N-dimethylamino)propyl zinc	Si wafers, polyethylene terephthalate	N/A	N/A	ZnO thin, uniform, smooth, stoichiometric, and highly transparent films	[27]
	MW reactor	optimal parameters: p = 30 mbar P = 60 W t = 90 min	80% Ar/ 20% O_2_	Ar/Zn(CH_3_)_2_ c_Zn(CH3)2_ = 1061 ppm	TEM grids	spherical	4.2–5.9 nm	band gap at 3.28 eV	[23]
	MW magnetron	t = 1 and 4 s	Air	Zn swarf collected from the drilling of the Zn ingot	Al foil, glass, paper, microfiber, polycarbonate film, paraffin wax	round at 1 s, nanowires at 4 s	ϕ 50 nm at 1 s, ϕ 65 nm at 4 s	wurtzite structure of ZnO, good crystallographic, quality of nanowires	[28]
	Pulsed high-current coaxial magnetoplasma accelerator plasma jet	Pulsed mode with:E_pulse_ = 27.7 kJ I = 10^5^ A t = 1 ms	O_2_	Metallic Zn electrode	Wall of reactor chamber	Hexagonal	Size 150–350 nm	Production of 10 g ZnO per 1 cycle, single-crystalline structure	[29]
	Coaxial magnetoplasma accelerator with zinc electrodes	U = 3.4 kV I = 100 kA W= 27.7 kJ	80% O_2_	Zn electrode in plasma	SPS-ceramics	round and hexagonal shape	10–150 nm	high purity, single-crystalline hexagonal ZnO	[7]
Atmospheric-pressure	Alternative/direct (AC/DC) current dielectric barrier discharge (DBD)	U = 16.7 kV f = 5 kHz t = 30–600 s (optimum 240 s)	Air	Zn plate	Zn plate	porous ZnO nanosheet-assembly film after calcination at 250°C for 1 h	30–50 nm	single-crystalline structure, band gap of 3.24 eV, 90% UV absorption	[30]
	AC (pulse) plasma jet	U = 511 V I = 125 mA E = 1.050 J/s t = 1 h d = 10 mm	Air	Zn nitrate (ZN), Zn chloride (ZC), Zn sulphate (ZS)	N/A	rods, pillar and, flower-like for ZN, flower-like and rods for ZC, layered and aggregated structures for ZS	from 400 × 200 to 1600 × 600 nm, or ϕ 500 nm	good crystallinity, high purity and fewer structural defects	[31]
	AC (sinusoidal) dielectric barrier discharge plasma	p = 1 bar U = 15 kV_pp_ f = 29 kHz	He	0.5, 1 and 2 M zinc acetate aqu.	Si wafer	spherical	90–150 nm	nanocomposites of crystalline nanoparticles and amorphous matrix	[32]
	DC pulsed plasma jet	U = 275 V t = 30 s	N_2_	10 mM zinc acetate spin coated on substrate	F-doped tin oxide glass substrates	polygonal Pt/ZnO composite nanoparticles	N/A	catalytic activities of Pt/ZnO, low charge transfer resistance, high exchange current density	[33]
	DC plasma torch	P_nominal_ = 55 kW	Ar as primary and H_2_ as secondary gas	0.2 M and 0.4 M Zn acetate (ZA), Zn nitrate (ZN)	Al plates	nanowires, nanorods for ZA, porous microstructures, nanorods and shell-like particles for ZN	N/A	well crystalline wurtzite ZnO, fair photocatalytic properties	[34]
	DC plasma torch	P = 70 kW	N_2_/Ar (1:1) and N_2_ as carrier gases	50 ppm Zn powders	None (powder collector)	with N_2_/Ar rod-like, with N_2_ spherical	100–200 nm with N_2_/Ar, 30 nm with N_2_	high purity, high crystal quality of the ZnO nanopowders, wurtzite phase ZnO	[35]
	Plasma jet	p = 1 bar I = 40 mA t = 10 min d = 5 mm	Air	0.05 M zinc nitrate aqu.	Particles formed in liquid	Spherical and cylindrical	2 and 3 nm	a number of particles had a hexagonal shape	[3]
	Plasma torch	p = 86.1 kPa P = 15 kW	Ar as carrier gas	Zn nitrate powder 50 μm	N/A	spherical	~57 nm	hexagonal wurtzite structure, UV emission band at 383 nm, excellent electric properties, 80% optically transmissive, photocatalytic properties	[36]
	Microwave (MW) waveguide plasma	f = 2.45 GHz	High-pure air (HPA), compressed air (CA), O_2_, and 20% O_2_/80%N_2_	10 µm Zn powder	Wall of quartz tube	nanowires and terapods with HPA, nanorods with CA and O_2_, tetrapods with O_2_/N_2_	diameter 29.7–626.5, length 256.5–5835.0 nm	single crystal ZnO nanowires of a hexagonal crystal structure, UV absorption band near 370 nm	[37]
